# Physician-Staffed Emergency Vehicle Crash: A Case Report

**DOI:** 10.7759/cureus.21027

**Published:** 2022-01-08

**Authors:** Tomohiro Abe, Katsuhiro Kanemaru, Katsutoshi Saito, Taichiro Ueda, Hidenobu Ochiai

**Affiliations:** 1 Department of Emergency and Critical Care Medicine, Faculty of Medicine, University of Miyazaki, Miyazaki, JPN; 2 Shock and Trauma Center, Nippon Medical School Chiba Hokusoh Hospital, Inzai, JPN

**Keywords:** health services administration, occupational accidents, occupational injuries, traffic accident, prehospital emergency care

## Abstract

Physician-staffed vehicles are widely operated in many countries. There is a paucity of literature regarding physician-staffed emergency vehicle accidents. On an evening in January 2016, at the request of the fire department, a physician-staffed vehicle was dispatched with two physicians, a nurse, and a driver from the base hospital to the scene of a patient with cardiopulmonary arrest. The vehicle ran with the alerting siren and warning lights. On its way, the vehicle struck a car and the mission was canceled. The patient was transported to another hospital by the ambulance staff only. No passengers were injured. One physician and a nurse examined the driver of the struck car and transported the driver to the base hospital by additional ambulance units. Because there were no manuals or guidelines, the staff responses were not systematic. After the repair of the crashed vehicle and preparation of operation manuals for two months, the physician-staffed vehicle returned to service, and it has worked without any accident since then.

The physician-staffed vehicle is of benefit to critical victims and it rarely crashes. When the vehicle is involved in an accident, it results in multiple victims as well as additional emergency demands. Warning lights and sirens in the dark at a four-point crossroads might increase the risk of crashing. Information influx from the emergency scenes might distract the physicians’ attention and put stress on the driver, leading to dangerous high-speed emergency driving. Educational training and manuals in each hospital and a nationwide framework regarding safety operations and accidents are needed.

## Introduction

Physician-staffed rapid response vehicles are widely operated in many countries [1,2]. These vehicles are mainly intended to improve the outcomes of patients of trauma, cardiac diseases, respiratory failure, and cardiopulmonary arrest through advanced medical interventions beyond the capabilities of emergency medical technicians (EMTs)/paramedics prior to hospital admission [3-7].

In Japan, EMTs can perform limited interventions for victims: endotracheal intubation, supraglottic airway insertion, defibrillation using an automated external defibrillator, peripheral venous catheter insertion, adrenaline administration for cardiopulmonary arrest, glucose administration for hypoglycemic coma, and fluid administration for hypovolemic shock. Thus, many hospitals in Japan operate physician-staffed rapid response vehicles after the revision of the road traffic law in 2008 [8]. These rapid response vehicles aim to deliver medical staff and equipment to the scene in order to provide advanced medical interventions that are beyond the EMTs’ competencies, but do not transport patients. Similar to police vehicles and ambulances, physician-staffed vehicles possess emergency warning lights and sirens to warn other drivers and pedestrians. These vehicles are entitled to the same privileges as other emergency vehicles. Unlike police cars or ambulances, there is no nationwide accident reporting system of the physician-staffed vehicles because these vehicles are independently operated by each hospital in Japan.

There are no prior reports of a physician-staffed vehicle crash, although several ambulance crashes have been reported [9]. Here we present the experience of a crash of a physician-staffed vehicle during an emergency mission. This article was previously presented as a meeting abstract at the 13th Japan Society for Prehospital Medicine Annual Scientific Meeting, on December 1, 2018.

## Case presentation

**Figure 1:**
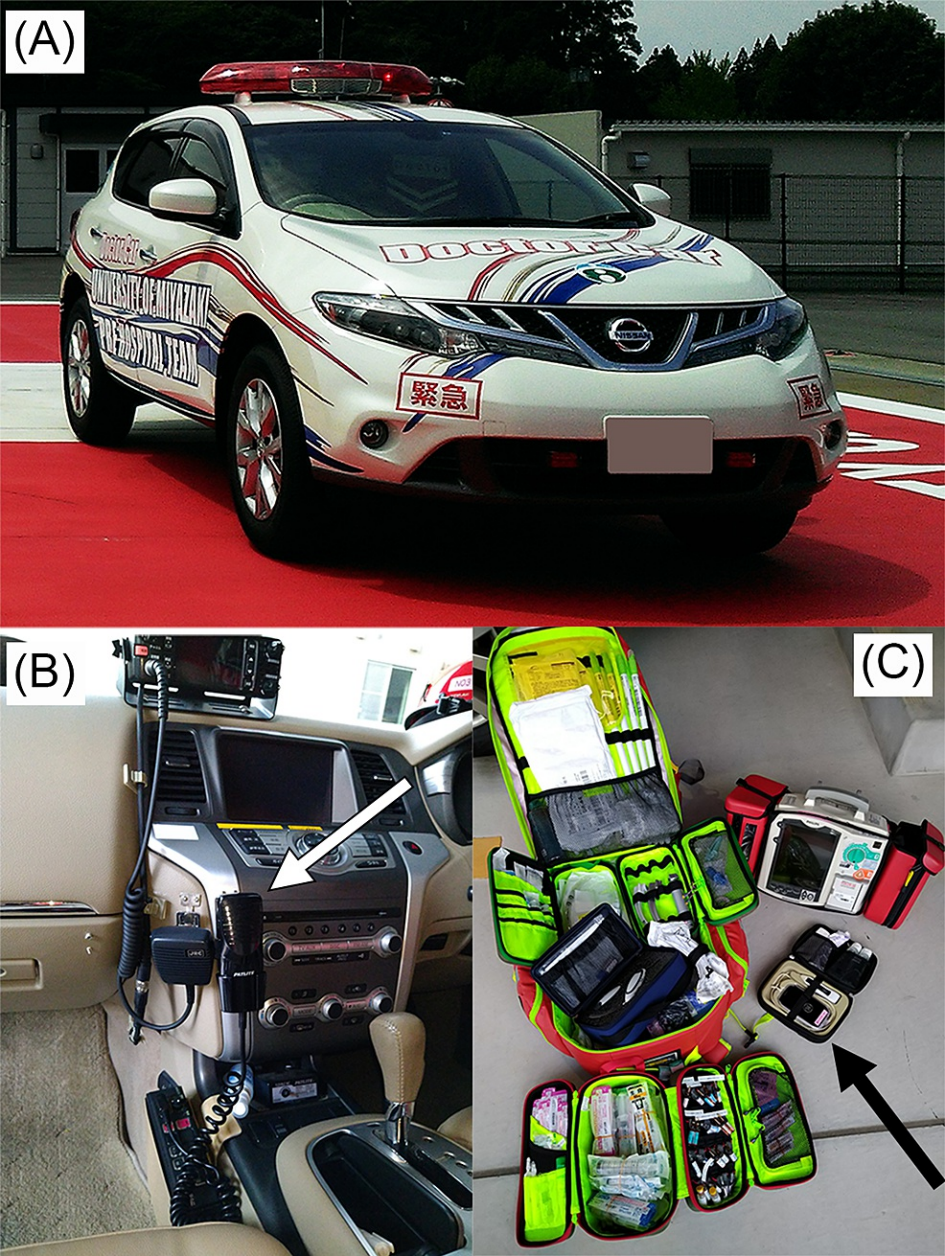
Physician-staffed vehicle (A), view from the seat of the chief physician (B), and medical equipment within the vehicle (C) (A) This vehicle is based on the Nissan Murano® and has been modified as an emergency vehicle. It is equipped with warning lights in front and on the roof. A siren and loudspeaker are attached to the front of the vehicle. Occupants can alert nearby vehicles via the loudspeaker. (B) The chief physician assists the driver by alerting nearby vehicles using the microphone (white arrow). (C) The vehicle carries medical equipment and medications to perform medical interventions, including administration of medication, surgical airway management, tube thoracotomy, resuscitative thoracotomy, pericardiocentesis, manual defibrillation, and percutaneous pacing. The vehicle also carries a portable ultrasound device (black arrow) for emergency ultrasound examination on scene.

The accident occurred 21 months after the introduction of the physician-staffed vehicle system at the University of Miyazaki Hospital (Figure 1).

**Figure 2:**
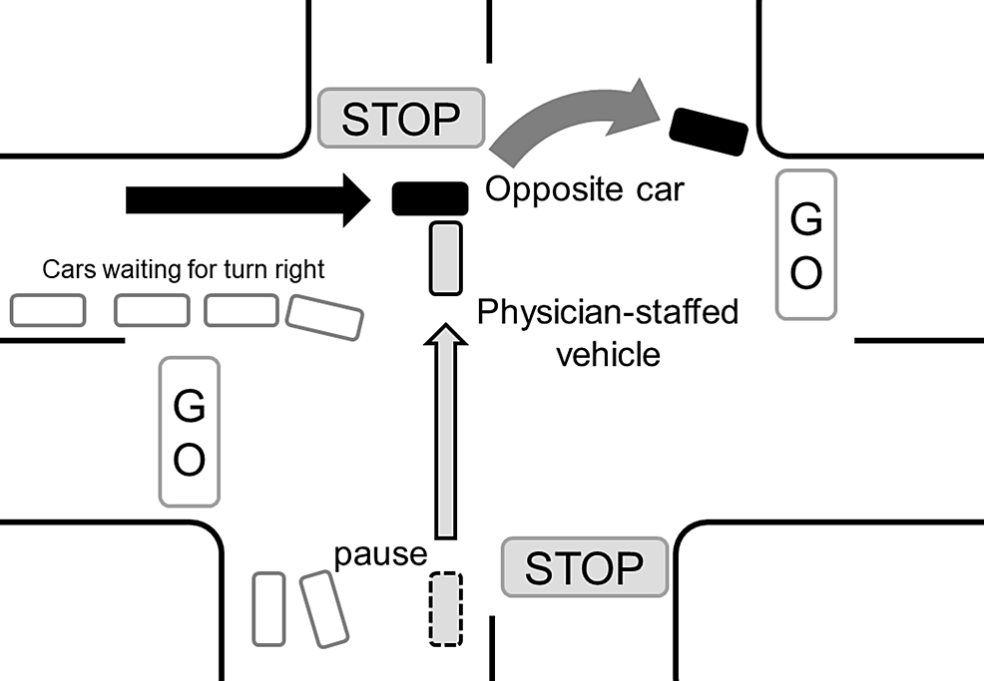
Diagram of the accident The physician-staffed vehicle (gray box) paused at the entrance of the crossroads signaling stop. When the vehicle proceeded straight to the crossroads, it struck a car (black box).

The fire department received a call that a man was suspected of experiencing cardiopulmonary arrest because of foreign body airway obstruction at 06:12 pm in January 2016. The fire department requested the hospital to dispatch the physician-staffed vehicle because the victim would need advanced cardiopulmonary life support with cricothyroidotomy. The vehicle was dispatched four minutes after the request with two physicians, one nurse, and a driver. The driver was a 60-year-old man with more than 30 years of emergency driving experience as a policeman. The car ran with a siren and warning lights, with the chief physician alerting nearby vehicles with a microphone en route. Approximately 15 minutes after dispatch, when the car was entering a crossroads with a stop signal, the chief physician received a phone call from the ambulance staff that was reaching the patient. Subsequently, when the car stopped and slowly (approximately 15 km/h) entered the crossroads, it struck another car that came from behind some waiting cars on the left side (Figure 2).

The chief physician communicated via radio to the fire department and the ambulance waiting for the response team that the car had crashed and could not continue the mission. The patient was treated by ambulance staff only. The patient had experienced cardiopulmonary arrest because of foreign body airway obstruction. The ambulance staff successfully performed endotracheal intubation but could not achieve peripheral venous catheter insertion. Subsequently, they transported the patient to the emergency room of the nearest hospital. The patient’s heartbeat was recovered at the emergency room, but he died due to severe hypoxic brain damage in a day.

At the scene of the accident, none of the staff members were injured. The driver of the struck vehicle was the only person injured in the accident. The driver was not seriously injured. The chief physician reported the accident to the base hospital to ask for instructions regarding the victim and the crashed car. At the base hospital, a physician and administrative staff dealt with the accident. Because no accident response manual was prepared, it was difficult for the staff to instruct the response team at the scene and respond officially, including asking to tow the car and reporting the accident to the insurance company.

Because there was no guidance regarding victim transportation, the driver of the struck vehicle was transported along with a physician and a nurse to the base hospital of the physician-staffed vehicle by additional ambulance units. On examination in the emergency room, the driver only had a bruise on the knees and the driver was discharged home. No significant injuries were found during the examination of all crew members. The damaged vehicle was towed to a repair facility at the request of the hospital.

**Figure 3:**
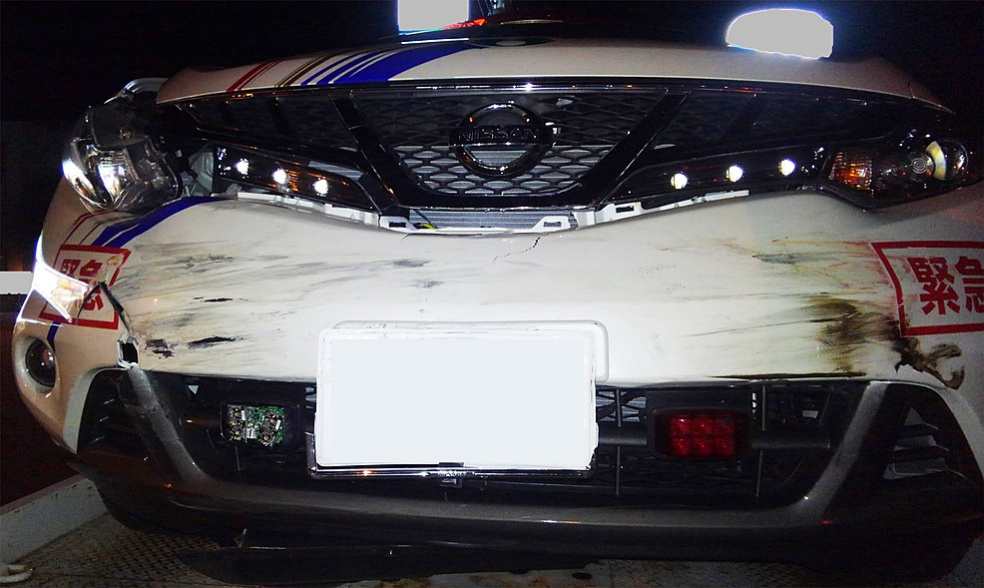
Damaged vehicle The bumpers were damaged. There was no damage to the wheels and cabin.

The physician-staffed car system had to be discontinued for two months because the vehicle needed repair (Figure 3).

During this period, we prepared an original accident manual and action cards for use at the base hospital and the scene. We summarized a list of emergency contacts, treatment and transport for victims involved in the accident, towing of the crashed vehicle, and on-site verification as a manual and action cards. We have also started daily staff education, including confirmation of safety operations. After the system started working again, we have conducted more than 500 missions (including more than 40 cases of cardiopulmonary arrest and 140 cases of trauma) without any accidents for more than five years.

## Discussion

Here we reported a case of an accident of a physician-staffed vehicle on the way to an emergency scene. The accident prevented physicians from reaching the patient and led to additional emergency needs.

The order of this accident reflects several features of an emergency vehicle accident. The emergency vehicle was moving straight and crashed into another vehicle at an angle, at a four-point crossroads with a stop signal, and in the dark [10]. Contrary to the expected avoidance of vehicle accidents, emergency driving with warning lights and a siren was associated with an increase in ambulance crashes [11,12]. In addition, many emergency vehicle crashes occurred due to a failure to yield the right-of-way [10]. Attention must be paid to nearby vehicles independent of the warning lights and sirens, especially in settings associated with the risk of emergency vehicle accidents. In our system, the chief physician seated in the front passenger seat assists the driver by alerting other vehicles and pedestrians using a microphone. At that time of the accident, the chief physician was attending to a phone call from the ambulance staff on the scene; thus, the physician was unavailable to alert nearby vehicles. Information from the destination scene would be useful for the physician to plan treatments earlier, but it may place secondary burdens on the physician [13].

From reports of rescue helicopter crashes by the National EMS Pilots Association, any pressure that makes the pilot take excessive effort can result in a crash [14]. This phenomenon would also apply to emergency vehicles because many severe accidents among emergency service vehicles occur during emergency missions, including response to the event and transportation [15,16]. Ironically, high-speed emergency driving saves little time in urban settings [17]. Time stress on the driver and associated emergency staff should be considered dangerous. Analysis shows that most ambulance crashes damage property and not occupants; however, the crash sometimes results in fatalities and is the leading cause of fatalities among emergency medical technicians [15]. Analysis has revealed that injuries and fatalities among emergency medical service occupants are higher than among other workers [16]. Sitting in the physician-staffed vehicle with high-speed emergency driving would be the most dangerous occupational setting for physicians. A culture of safety and education regarding emergency driving for all occupants is essential. Moreover, the vehicles should be tough enough to withstand crashes, even though in Japan, the vehicle is only required to be equipped with sirens and warning lights.

Our system did not possess any manuals on accidents before the crash. In Japan, the physician-staffed vehicles are operated by each hospital; the hospitals must deal with the accidents themselves. When an accident occurs, the scene staff must deal with multiple problems, such as the victims of the original case for which the vehicle was dispatched, victims on the opposing vehicles, crashed cars, and their own injured staff. Manuals on such accidents should be prepared by each hospital.

Training for the drivers and proactive risk management is effective in reducing emergency vehicle accidents [18]. Gathering and analysing the data of the physician-staffed vehicle accident are necessary to identify risks, although this is not currently done in Japan. Thus, a nationwide systematic framework for physician-staffed vehicles is needed that establishes safety requirements for the vehicle, gathers accident data, analyses it, and issues standardized safety operation guidelines.

## Conclusions

Here we presented a case involving a physician-staffed vehicle accident. Physician-staffed vehicle accidents create more casualties and place additional demands on the fire department. For safe physician-staffed vehicle operations, educational courses and manuals regarding safe operation and accidents should be established by each hospital. A nationwide systematic framework is needed to establish standard guidelines.
